# Cation induced differential effect on structural and functional properties of *Mycobacterium tuberculosis *α-Isopropylmalate synthase

**DOI:** 10.1186/1472-6807-7-39

**Published:** 2007-06-19

**Authors:** Kulwant Singh, Vinod Bhakuni

**Affiliations:** 1 Division of Molecular and Structural Biology, Central Drug Research Institute, Lucknow-226 001, India (CDRI communication number-7085)

## Abstract

**Background:**

α-isopropylmalate synthase (MtαIPMS), an enzyme that catalyzes the first committed step of the leucine biosynthetic pathway of *Mycobacterium tuberculosis *is a potential drug target for the anti-tuberculosis drugs. Cations induce differential effect of activation and inhibition of MtαIPMS. To date no concrete mechanism for such an opposite effect of similarly charged cations on the functional activity of enzyme has been presented.

**Results:**

Effect of cations on the structure and function of the MtαIPMS has been studied in detail. The studies for the first time demonstrate that different cations interact specifically at different sites in the enzyme and modulate the enzyme structure differentially. The inhibitors Zn^2+ ^and Cd^2+ ^ions interact directly with the catalytic domain of the enzyme and induce unfolding/denaturation of the domain. The activator K^+ ^also interacts with the catalytic TIM barrel domain however, it does not induce any significant effect on the enzyme structure. Studies with isolated catalytic TIM barrel domain showed that it can carry out the catalytic function on its own but probably requires the non-catalytic C-terminal domain for optimum functioning. An important observation was that divalent cations induce significant interaction between the regulatory and the catalytic domain of MtαIPMS thus inducing structural cooperativity in the enzyme. This divalent cation induced structural cooperativity might result in modulation of activity of the catalytic domain by regulatory domain.

**Conclusion:**

The studies for the first time demonstrate that different cations bind at different sites in the enzyme leading to their differential effects on the structure and functional activity of the enzyme.

## Background

Tuberculosis is the second leading infectious cause of mortality worldwide. *Mycobacterium tuberculosis *remains one of mankind's deadliest pathogen, responsible for approximately two billion deaths worldwide every-year, which is one-third of the world's population [1]. Although effective drugs against tuberculosis exist, therapy requires prolonged treatment with several drugs, leading to problems in compliance and emergence of multidrug resistance [2]. There is an urgent need for more effective drugs against tuberculosis. Hence, development of new drugs and characterization of new targets is urgently required.

Mycobacteria synthesize the branched-chain amino acids, L-valine, L-leucine and pantothenic acid from α-ketoisovalerate (α-KIV). The essentiality of this pathway in *M. tuberculosis*, and its absence in humans makes the enzymes of this pathway attractive target/s for development of drug/s for treatment of tuberculosis [3]. The first step in the L-leucine biosynthesis is the formation of α-isopropylmalate from acetyl-CoA and α-KIV that is catalyzed by α-isopropylmalate synthase (α-IPMS). α-IPMS is an allosteric enzyme that is present in various organisms like bacteria, fungi and plants.

Crystal structure of only one α-IPMS, MtαIPMS a dimeric enzyme, has been reported to date [4]. Each monomer of the enzyme is folded into two major N- and C-terminal domains which are separated by two small sub-domains, sub domain I and sub domain II that are joined by a flexible hinge [4]. The N-terminal domain contains the active site and the C-terminal domain the L-leucine binding site of the enzyme. The functional characterization of α-IPMS from several organisms have been reported [5-8]. They share some common features like requirement of monovalent cations for activity, feedback inhibition by L-leucine and narrow substrate specificity for analogues of α-KIV. The kinetic parameters of the substrates for MtαIPMS are significantly influenced by cations both monovalent and divalent. The K^+ ^is physiological activator of the enzyme [9,10]. Divalent cations show broad specificity for the functional activity of the enzyme. Mg^2+ ^and Mn^2+ ^induce activation whereas; Zn^2+ ^and Cd^2+ ^induce inhibition of the functional activity of the enzyme [9]. The kinetics of activation or inhibition of the MtαIPMS by the cations has been extensively studied and possible mechanisms have been proposed. However, no experimental validations of the proposed mechanism/s have yet been documented.

In order to understand the mechanism of modulation of functional activity of MtαIPMS by cations we have carried out detailed functional and structural studies. For studying the specificity of interaction of cations with different domains of the enzyme, the catalytic TIM barrel domain was isolated and purified. The effects of cations on the structural and functional properties of the isolated TIM barrel domain were carried out. Comparative analysis of the effect of cations on the isolated catalytic domain and the full-length enzyme provides intriguing insight into the possible mechanism of cation induced changes in the MtαIPMS.

## Results

### Over-expression and purification of MtαIPMS

**Figure 1:**
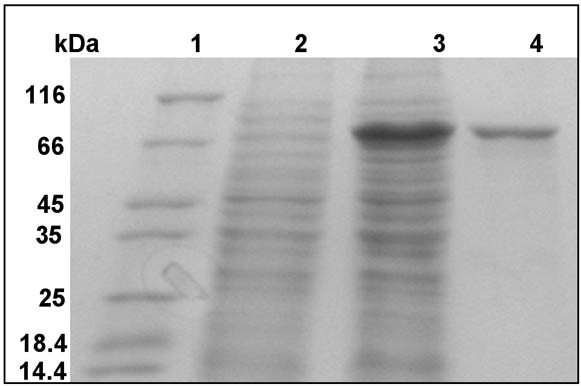
**Over-expression and purification of MtαIPMS**. SDS-PAGE analysis of cell lysate over-expressing MtαIPMS and purified protein. Lanes 1–4 represent molecular weight markers, supernatant of un-induced cell lysate, supernatant of induced cell lysate and purified protein, respectively.

The expression of the recombinant MtαIPMS was good and the expressed protein was present predominantly (>90%) in the soluble fraction (Figure 1). The purified protein was homogenous as indicated by a single protein band on SDS-PAGE^1 ^(Figure 1) and a single peak in ESI-MS of molecular mass about 73.1 kDa (data not shown).

### Differential effect of metal cations on the functional activity of MtαIPMS

**Figure 2:**
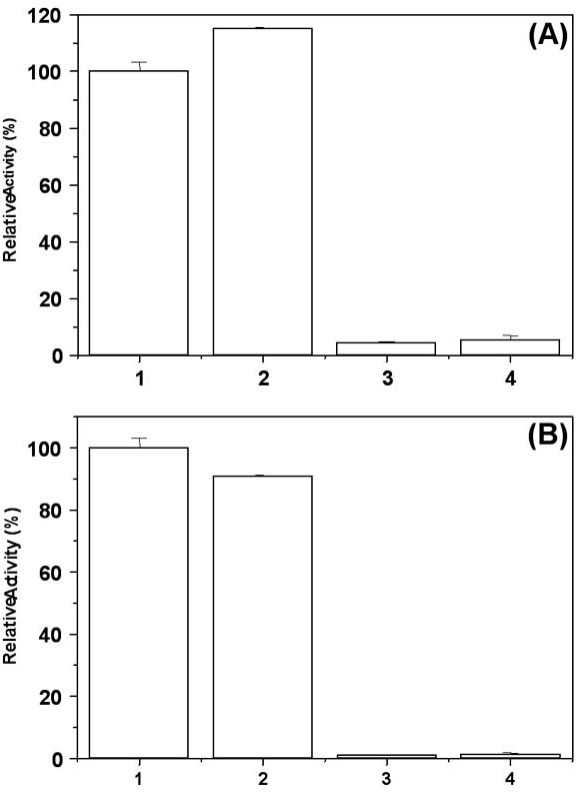
**Cations influence enzymatic activity of both MtαIPMS and the TIM barrel domain**. Effect of KCl, MgCl_2_, ZnCl_2 _or CdCl_2 _on enzymatic activity of MtαIPMS (Panel A) and the catalytic TIM barrel Domain (Panel B). In both the panels the bars 1 to 4 represent samples of protein in presence of 20 mM KCl, 20 mM KCl + 5 mM MgCl_2_, 20 mM KCl + 5 mM MgCl_2 _+2 mM ZnCl_2 _and 20 mM KCl + 5 mM MgCl_2 _+ 2 mM CdCl_2_, respectively. The data is represented as percent activity with the activity of the protein in presence of 20 mM KCl taken as 100%. The data is represented as mean ± SD of three values.

Metal cations, both monovalent and divalent, showed broad specificity on the functional activity of MtαIPMS (Figure 2A). Monovalent cation K^+^, was essential for the functional activity of the enzyme as in its absence no activity was observed. Presence of Mg^2+ ^ions along with K^+ ^resulted in enhancement (about 115%) of functional activity. Zn^2+ ^and Cd^2+ ^ions induced significant inhibition of above 90% of the functional activity of MtαIPMS. These observations are similar to the earlier report [9].

### Cations induce differential effects on the secondary structure of MtαIPMS

**Figure 3:**
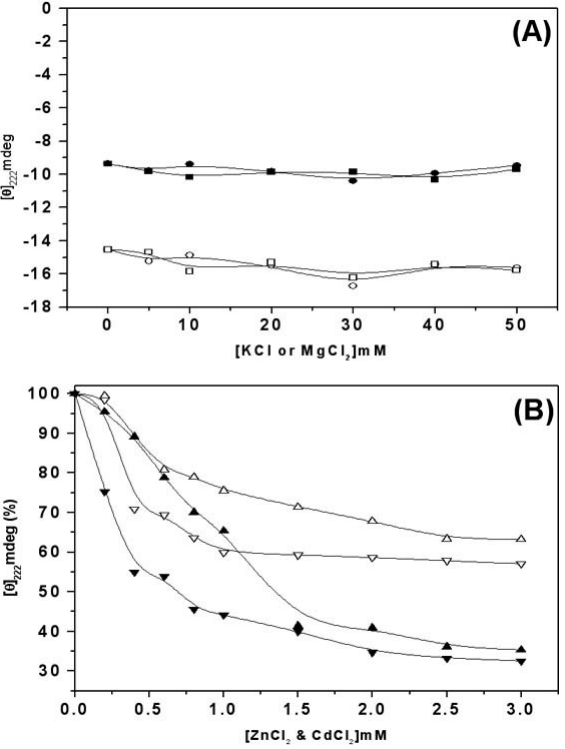
**Divalent cations have differential effects on the secondary structure of MtαIPMS and TIM barrel domain**. A. Effect of increasing concentration of KCl (circle) and MgCl_2 _(square) on the CD ellipticity at 222 nm of MtαIPMS (open symbols) and TIM barrel domain (closed symbol). B. Effect of increasing concentration of ZnCl_2 _(up triangle) and CdCl_2 _(down triangle) on the CD ellipticity at 222 nm of MtαIPMS (open symbols) and TIM barrel domain (closed symbols).

The effect of cations on the structure of MtαIPMS was studied by monitoring changes in the far-UV CD of enzyme at 222 nm in presence of increasing concentration of MgCl_2_, KCl, ZnCl_2 _and CdCl_2_(Figure 3). No significant alteration in the secondary structure of the enzyme was observed in presence of MgCl_2 _and KCl concentration even up to 50 mM (Figure 3A). ZnCl_2 _and CdCl_2 _induced significant loss of secondary structure of MtαIPMS. An exponential loss of CD ellipticity at 222 nm was observed with increasing concentration of ZnCl_2 _or CdCl_2_. However, a maximum loss of about 30 and 40 percent of CD signal was observed with about 2 mM CdCl_2 _and ZnCl_2_, respectively (Figure 3B). This suggests that interaction of Cd^2+ ^and Zn^2+ ^ions with MtαIPMS leads to only partial loss of secondary structure, i.e partial denaturation of the enzyme.

**Figure 4:**
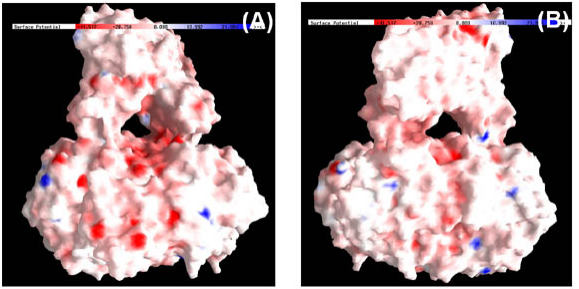
**Electrostatic potential of molecular surface of MtαIPMS dimer**. The colors blue and red represents negative and positive potential. Panels A and B represent MtαIPMS dimer molecule in two different orientations. The molecular surface was displayed using GRASP.

We wanted to understand the underlying mechanism for differential action of cations on both the structure and function of the MtαIPMS. The electrostatic surface potential of the enzyme as obtained from GRASP shows no large concentration of positive or negative charged regions on the enzyme surface (Figure 4). Hence, non-specific binding of cations on protein surface will be low. Furthermore, a differential effect of activation or inhibition and partial unfolding or no effect on the structure of enzyme by similarly charged cations like Mg^2+^, Cd^2+ ^and Zn^2+ ^was observed. These observations suggest a possibility of interaction of cations with similar charges at different sites in the enzyme. This possibility is further strengthened by the observation that Cd^2+ ^and Zn^2+ ^induce only partial unfolding but almost complete loss of functional activity of the enzyme. So we concentrated our efforts on mapping site of interaction of various cations on MtαIPMS. To obtain an isolated folded catalytic domain of the enzyme, limited proteolysis technique was applied which has been demonstrated by us to be a technique of choice for obtaining isolated domain/s in folded functionally active form from the full-length protein [11-13].

### Identification of a structural domain of MtαIPMS

**Figure 5:**
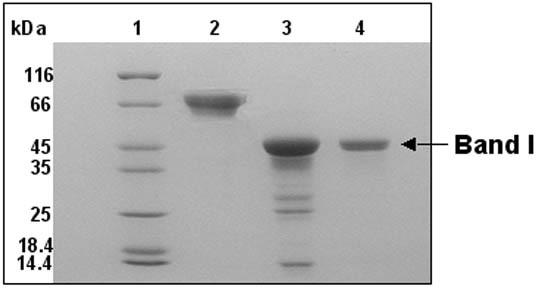
**Limited proteolysis of MtαIPMS with α-chymotrypsin at pH 7.5 and 25°C**. SDS-PAGE profile of the protein fragment(s) obtained on limited proteolysis of recombinant MtαIPMS with α-chymotrypsin. Lanes 1–4 represent molecular weight markers, undigested MtαIPMS, protein digested with α-chymotrypsin and purified Band I, respectively.

Figure 5 summarizes the SDS-PAGE profile of the protein fragments obtained on limited proteolysis of recombinant MtαIPMS with α-chymotrypsin. A major band (Band I) along with three minor bands of lower molecular weight proteins/fragments were observed. The Band I purified by SEC^1 ^showed a molecular mass of 46.6 kDa (as determined by ESI-MS). There are about 47 cleavage sites for α-chymotrypsin spread all over the primary sequence of MtαIPMS however, on limited proteolysis, an intact domain of about 46 kDa was obtained. This suggests that the 46 kDa domain obtained on limited proteolysis of full length protein is in a folded conformation as several of the proteolytic sites present in it are buried in protein interior and not accessible to protease for cleavage under experimental conditions.

**Figure 6:**
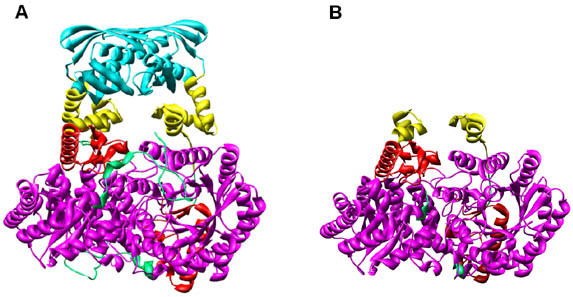
**MOLSCRIPT model of MtαIPMS (Panel A) and the TIM barrel domain (Panel B)**. The TIM barrel domain (magenta), sub-domain I (yellow), sub-domain II (red) and regulatory domain (cyan). The model has been generated using program UCSF Chimera.

The Band I showed no affinity for the nickel-nitrilotriacetic acid-agarose matrix and anti-His antibody on western (Data not shown) suggesting that it corresponds to portion of MtαIPMS in which N-terminus has been removed as the histidine tag is attached to the N-terminus of the full-length protein. For determining the site of cleavage of protease in the protein, N-terminal sequencing of the obtained fragment was carried out. It had a sequence RPFAE. Based on N-terminal sequence and the observed molecular mass, Band I corresponds to the protein fragment Arg47 to Phe457 of the MtαIPMS. This fragment consists of the TIM barrel portion, sub domain I and a small part of sub domain II of the full-length enzyme (Figure 6). It has been designated as the TIM barrel domain in this study.

### The TIM barrel domain of MtαIPMS is stabilized as a functionally active dimer

**Figure 7:**
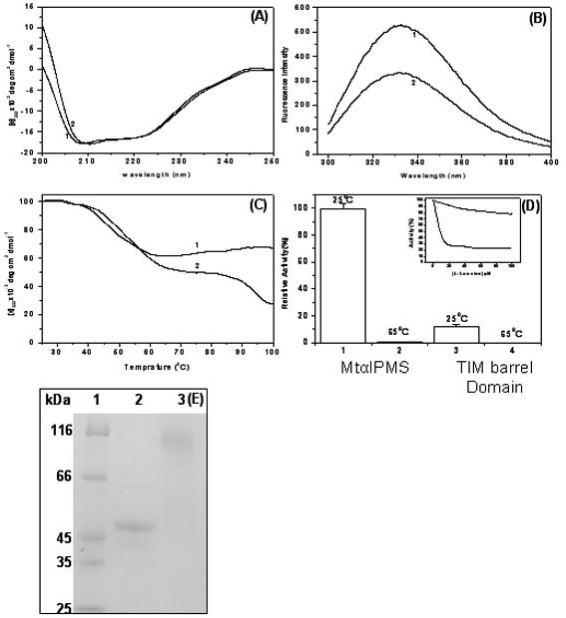
**Structural and functional properties of MtαIPMS and the TIM barrel domain**. **A**. Far-UV CD spectra of MtαIPMS (profile 1) and the TIM barrel domain (profile 2). **B**. Tryptophan emission spectra of MtαIPMS (profile 1) and the TIM barrel domain (profile 2). **C**. Thermal unfolding of MtαIPMS and the TIM barrel domain. Effect of increasing temperature on the CD ellipticity at 222 nm of MtαIPMS (profile 1) and the TIM barrel domain (profile 2), respectively. The values have been represented as percentage with the value obtained at 25°C for each sample taken as 100 percent, respectively. **D**. Enzymatic activity of MtαIPMS and the TIM barrel domain. Relative enzymatic activity of MtαIPMS (Bar 1 and 2) and the TIM barrel domain (Bar 3 and 4) at 25 and 65°C, respectively. The values have been represented as percent activity with the value observed for MtαIPMS at 25°C taken as 100%. The data is represented as mean ± SD of three values. Inset shows the loss of enzymatic activity of the MtαIPMS (closed symbols) and the TIM barrel domain (open symbols) on incubation with increasing concentration of L-lecucine. The data has been represented as percentage with the activity of MtαIPMS or the TIM barrel domain in absence of Leucine taken as 100 percent. **E**. SDS-PAGE profile of glutaraldehyde cross-linked TIM barrel domain. Lanes 1–3 represent molecular weight markers, uncrosslinked and glutaraldehyde cross-linked TIM barrel domain, respectively.

To confirm that the isolated TIM barrel domain is stabilized in folded conformation, structural studies were carried out. Figure 7A and 7B, shows far UV-CD and tryptophan fluorescence spectra respectively, of the TIM barrel domain. A far-UV profile typical of a αβ-protein [14] was observed. The tryptophan fluorescence emission spectra of the domain showed emission wavelength maxima at about 334 nm suggesting that the tryptophan moieties in the TIM barrel domain are buried in the hydrophobic core of the protein [15]. These observations support the suggestion that the isolated TIM barrel domain is stabilized in folded conformation under physiological conditions.

The most convincing result proving that the isolated TIM barrel domain is stabilized in folded conformation similar to that in which it is present in the full-length protein came from thermal denaturation studies. Figure 7C shows the comparative thermal denaturation profile of native MtαIPMS and isolated TIM barrel domain as studied by monitoring the loss of CD signal at 222 nm at increasing temperature. For MtαIPMS at pH 7.5, a single sigmoidal transition centered at about 49°C and corresponding to loss of only about 35% of the CD signal was observed. For the TIM barrel domain, a biphasic transition with a main transition having Tm about 52°C corresponding to loss of about 50% structure of the protein along with a minor transition with Tm about 92°C and loss of about 70% CD signal was observed. The similar thermal denaturation profile for the main transition of TIM barrel domain and the heat sensitive domain of the MtαIPMS indicates that they correspond to same portion of the full-length protein i.e. the catalytic N-terminal domain. This is confirmed by complete loss of functional activity of both the full-length enzyme and isolated TIM barrel domain at 65°C (Figure 7D) suggesting that it is indeed the catalytic domain that is unfolded under these conditions. These observations conclusively demonstrate that isolated catalytic TIM barrel domain is stabilized in a conformation similar to that in, which it is present in native conformation in the full-length protein.

The quaternary structure of the isolated TIM barrel domain was obtained by SDS-PAGE analysis of glutaraldehyde crosslinked sample, Figure 7E. A molecular mass of about 93 kD was observed for the cross-linked sample, which is about twice the mass of about 46.6 kDa observed for this fragment. Hence, isolated TIM barrel domain is stabilized as a dimer under physiological conditions.

According to crystal structure, TIM barrel domain of MtαIPMS is the catalytic domain and it contains all the residues required for the catalytic activity of enzyme [4] so we carried out a functional activity assay on it. The TIM barrel domain did show functional activity however; it was only about 12% of that observed for the MtαIPMS (Figure 7D). Furthermore, unlike MtαIPMS whose functional activity was significantly inhibition by L-leucine, the functional activity of TIM barrel domain was not affected by L-leucine (Figure 7D, inset). X-ray studies have demonstrated that L-leucine binds to the regulatory domain of the enzyme [4][16,17] which is absent in the isolated TIM barrel domain, explaining why L-leucine did not inhibit the activity of the catalytic domain. These results demonstrate that the TIM barrel domain obtained on limited proteolysis of the MtαIPMS with α-chymotrypsin is indeed the catalytic domain of the enzyme and it can carry out the catalytic function on its own.

### Differential effect of metal cations on the functional activity and structural properties of isolated TIM barrel domain of MtαIPMS

Figure 2B summarizes the effect of KCl, MgCl_2_, ZnCl_2 _and CdCl_2 _on functional activity of TIM barrel domain. Monovalent cation K^+^, was found to be essential for functional activity as in its absence no activity was observed (Figure 2A). The Mg^2+ ^ions did not show any further enhancement in activity observed in presence of K^+ ^ions. The Zn^2+ ^and Cd^2+ ^ions showed inhibition of functional activity of the isolated TIM barrel domain with almost complete loss observed at about 3 mM ZnCl_2 _or CdCl_2_.

Figure 3 shows the changes in the far-UV CD of the TIM barrel domain at 222 nm in presence of increasing concentration of MgCl_2_, KCl, ZnCl_2 _or CdCl_2_. The addition of MgCl_2 _or KCl, even up to 50 mM concentration, gave no significant alteration in the secondary structure of the protein (Figure 3B). However, with increasing concentration of ZnCl_2 _or CdCl_2 _an exponential loss of CD signal at 222 nm was observed and at about 2 mM ZnCl_2 _or CdCl_2_, about 70 percent loss of secondary structure of the TIM barrel domain was observed.

### Divalent cations induce structural cooperativity in the MtαIPMS

**Figure 8:**
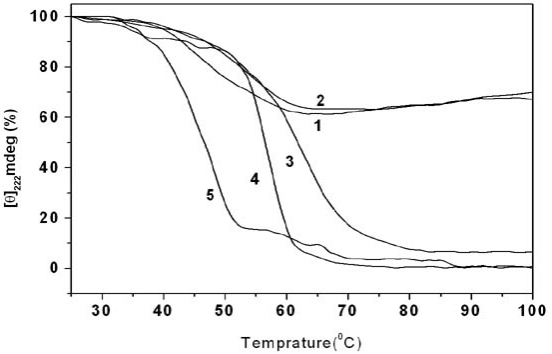
**Thermal unfolding of salt-treated MtαIPMS**. Effect of increasing temperature on the CD ellipticity at 222 nm of MtαIPMS (profile 1), MtαIMPS in presence of 5 mM KCl (profile 2), 5 mM MgCl_2 _(profile 3), 0.25 mM ZnCl_2 _(profile 4) and 0.25 mM CdCl_2 _(profile 5), respectively. The values have been represented as percentage with the value obtained at 25°C for each sample taken as 100 percent, respectively.

To analyze whether the two structural domains, N-terminal or TIM barrel domain and the C-terminal or regulatory domain, of MtαIPMS interact strongly with each other or they are independent folding/unfolding units of the protein, thermal denaturation studies were carried out by monitoring the loss of secondary structure of enzyme at increasing temperatures. Figure 8 shows the changes in CD ellipticity at 222 nm for MtαIPMS as a function of temperature. A broad sigmoidal transition with an apparent Tm of about 49°C was observed. An interesting observation was that a loss of only about 35% CD ellipticity at 222 nm was associated with the thermal denaturation of MtαIPMS, demonstrating that the major part of the protein molecule is resistant to thermal unfolding. This indicates that MtαIPMS is composed of two unfolding units that behave independently and have different thermal stabilities: one being sensitive to thermal denaturation and the other resistant to it. A similar sensitivity of structural domains to thermal denaturation has been observed for other proteins [13]. Hence, full-length MtαIPMS is structurally a non-cooperative molecule having two distinct structural units that fold/unfold independently of each other.

The effect of cations on the thermal denaturation of MtαIPMS was studied by incubating the protein with salts like KCl, MgCl_2_, CdCl_2_or ZnCl_2_. As all these salts contain the same anion (i.e. Cl^-^), the different effects observed in the comparative study using these salts will be mainly due to different cations. Figure 8 summarizes the thermal denaturation profiles of KCl-, MgCl_2_-, CdCl_2_-_, _ZnCl_2_- or pH 7.5-incubated MtαIPMS. KCl-treated MtαIPMS showed a thermal denaturation profile similar to that observed for the enzyme in absence of salt demonstrating that in presence of KCl the enzyme still behaves non-cooperatively. However, for MgCl_2_-, CdCl_2_- or ZnCl_2_-treated MtαIPMS, a single transition with almost complete loss of secondary structure was observed. This demonstrates that divalent cations induce strong interactions between the structural domains of enzyme resulting in induction of cooperativity in the otherwise non-cooperative MtαIPMS molecule. An interesting observation was that the Tm associated with the CdCl_2_-treated MtαIPMS (about 49°C) is significantly lower than that of 65°C observed for MgCl_2_-treated MtαIPMS. These differences in Tm in the presence of different salts are due to the differential effect of salts on the enzyme structure. The CdCl_2 _induces partial denaturation of the enzyme whereas, the MgCl_2 _does not affect the structure of enzyme significantly. The information that divalent cations induce co-operativity in the otherwise non-cooperative MtαIPMS molecule is of importance as it suggests that Mg^2+ ^ions that are included in the enzyme assay induce interaction between the catalytic N-terminal and the regulatory C-terminal domain as a result of which the regulatory domain can modulate the activity of the catalytic domain.

## Discussion

MtαIPMS in the functional form, is an intimately associated extended dimer [4]. The dimer interface of the enzyme covers only the N- and C-terminal domains which are in contact with each other in the native conformation of the enzyme [4]. The two TIM barrels in the dimer of enzyme pack against one another and are tied together by N-and C-terminal extensions. The N-terminal residues 18–50, from one monomer wind over the surface of the other monomer. The C-terminal domain of the enzyme also dimerizes. The TIM barrel without the N and the C-terminal extension contribute only 12 % of the total buried surface as compared to 80% of the total buried surface area attributed by the dimerization of the barrel including the N- and C-terminal extensions [4]. It has been demonstrated in this paper that the isolated TIM barrel domain (Arg47 to Phe457) that is devoid of the N-terminal extension and the C-terminal domain, is stabilized as a functionally active dimer. This suggests that the N-terminal extension and the regulatory C-terminal domain are not essential for the stabilization of the dimeric configuration of MtαIPMS under physiological conditions, which has also been indicated earlier [4].

The functional activity of the MtαIPMS is subject to major controls by small molecules. Metal cations, K^+ ^and Mg^2+ ^are the activators of MtαIPMS and their presence is important for the activity of the enzyme [9]. The activating molecules influence the oligomeric structure or conformation of the enzyme belonging to the family of Claisen-condensing enzymes, including several αIPMS [18-20]. However, our studies and a recent report [10] demonstrates that for MtαIPMS, both the activators K^+ ^and Mg^2+^, have no significant effect on the oligomeric or the secondary structure of the enzyme. In contrast, Zn^2+ ^and Cd^2+ ^induced partial and almost complete denaturation of the MtαIPMS and the catalytic TIM barrel domain, respectively. However, complete loss of enzymatic activity was observed for both the full-length enzyme and isolated catalytic domain under these conditions. These observations suggest that Zn^2+ ^and Cd^2+ ^bind directly to the catalytic domain of the enzyme leading to its unfolding/denaturation resulting in loss of activity. Binding of Zn^2+ ^ions leading to unfolding of the protein has recently been reported for spermadhesion PSP-I [21]. The activator K^+ ^ions induces activation of both the full-length enzyme as well as the isolated catalytic TIM barrel domain suggesting that it interacts directly with the catalytic domain and induce activation of the enzyme. These results for the first time demonstrate that different cations bind at different sites in the MtαIPMS resulting in differential modulation of functional and/or structural property of the enzyme.

One important observation was that the isolated TIM barrel domain showed only 12% activity of that observed for the MtαIPMS although the structural and stability studies showed that the isolated domain has similar conformation as that in which it is present in the native MtαIPMS. This suggests the possibility that probably the C-terminal domain of enzyme is necessary for optimum activity of the enzyme. In other words the non-catalytic C-terminal domain modulates the activity of the catalytic TIM barrel domain and its absence results in decrease in activity of the catalytic domain. This possibility is supported by the fact that L-leucine, which binds in the C-terminal domain, inhibits the activity of the MtαIPMS.

An important property of MtαIPMS found to be influenced by the cations is the structural cooperativity of the enzyme molecule. Divalent cations were found to induce cooperativity in the otherwise structurally non-cooperative MtαIPMS molecule. The induction of cooperativity in the MtαIPMS molecule by the Mg^2+^, which is an activator of the enzyme and is added to the reaction mixture during activity measurements, may help in modulation of functional activity of the TIM barrel domain by the regulatory domain.

## Conclusion

Comparative studies on the effect of cations on the structural and functional properties of MtαIPMS and its isolated catalytic TIM barrel domain demonstrates that different cations interact at different sites in the enzyme molecule inducing different effects on the enzyme structure. Zn^2+ ^and Cd^2+ ^ions interact with the catalytic domain of the enzyme and induce unfolding/denaturation of the domain and loss of its functional activity. The physiological activator K^+ ^ion also interacts with the catalytic domain and activates the enzyme however; it does not induce any significant structural change in the enzyme. The divalent cations were found to induce structural cooperativity in the otherwise non-cooperative MtαIPMS molecule. The study provides an intriguing insight into the structural details of the enzyme MtαIPMS and its regulation by the cations. This information will be of significant importance in designing of the inhibitors of the enzyme.

## Methods

### Materials

All chemicals used in the study were purchased from Sigma-Aldrich Chemical Company St. Louis, USA, and were of highest purity available. All chromatographic columns were purchased from GE Healthcare Biosciences, with the exception of Ni-NTA agarose and chelex-100 that were purchased from Qiagen and BioRad, respectively.

### Overproduction and purification of MtαIPMS

The overproduction and purification of recombinant MtαIPMS was carried out with slight modification of the earlier described method [22]. MtαIPMS was overproduced in *E. coli *C41 harboring the plasmid pProEX-HTa. Cells were grown in 2XYT medium with 100 mg/litre ampicillin and induced in mid log phase with 0.5 mM IPTG for 15 hours at 16°C for the synthesis of MtαIPMS. The overexpressed protein was purified by earlier reported procedure [22]. The purified protein showed >95% purity, as observed by ESI-MS and SDS-PAGE analysis, and was stored in 50 mM HEPES pH 7.5. Purified protein and all the buffers used in the study were subjected to divalent metal removal using chelex-100 resin.

### Assay for enzymatic activity

The enzymatic activity of MtαIPMS and TIM barrel domain were determined using 4,4'-dithiodipyridine (DTP) to detect the formation of coenzyme A (CoA) at 324 nm (ε = 19800 M^-1^cm^-1^) at 25°C as reported earlier [23]. A typical reaction mixture contained 50 mM HEPES (pH 7.5), 20 mM KCl, 5 mM MgCl_2, _100 μM DTP, 300 μM AcCoA, 300 μM α-KIV and 20 nM of MtαIPMS or TIM barrel domain. Reaction mixtures were incubated for 4 h at 25°C with salts and reactions were initiated by addition of α-KIV. For studies with isolated TIM barrel domain higher concentrations of AcCoA and α-KIV was also tried but no significant change in the measured enzymatic activity was observed.

To probe the effect of thermal changes on the full-length enzyme and the isolated TIM barrel domain, samples were heated for 15 min at the desired temperature before measurements were made.

### ESI-MS

The mass spectra were recorded on a MICRO-MASS QUATTRO II mass spectrometer (Micromass, Altricem, United Kingdom) equipped with an electrospray ionization (ESI) ion source as describe earlier [24].

### Limited proteolysis and purification of TIM barrel domain

2.0 mg/ml protein was subjected to limited proteolysis with α-chymotrypsin, at a protease to protein ratio of 1:50 (w/w) for 1 h at 25°C. The reaction was stopped by addition of phenylmethylsulfonyl fluoride to a final concentration of 1 mM, and the samples were analyzed on 10% SDS-PAGE. For purification of TIM barrel domain, proteolysed samples were loaded on the Superdex 200 HR 10/300 column interfaced with AKTA FPLC (GE Healthcare Biosciences). The column was pre-equilibrated and run with 50 mM HEPES, pH 7.5. 500 μl of the sample was injected in the column and run at 25°C at a flow rate of 0.3 ml/min, with detection at 280 nm.

### Fluorescence spectroscopy

Fluorescence spectra were recorded with Perkin-Elmer LS 50B spectroluminescencemeter in a 5 mm path length quartz cell. Protein concentration was 0.75 μM for all experiments and the measurements were carried out at 25°C. For monitoring tryptophan fluorescence the excitation wavelength of 280 nm was used and the spectra were recorded between 300 to 400 nm.

### Circular dichroism measurements

CD measurements were made with a Jasco J810 spectropolarimeter calibrated with ammonium (+)-10-camphorsulfonate. The results are expressed as the mean residual ellipticity (θ), which is defined as (θ) = 100 × θ_obs_/(lc), where θ_obs _is the observed ellipticity in degrees, c is the concentration in mol residue per litre, and l is the length of the light path in centimeters. The CD spectra were measured at an enzyme concentration of 0.75 μM with a 1 mm cell at 25°C. The values obtained were normalized by subtracting the baseline recorded for the buffer having the same concentration of salt under similar conditions.

### Metal ion induced structural alterations

For studying the cation induced structural alterations, protein (0.75 μM), except in case of ZnCl_2 _and CdCl_2 _(0.5 μM of protein), in 5 mM HEPES (pH 7.5) was incubated in the presence/absence of increasing concentration of KCl, MgCl_2, _ZnCl_2 _and CdCl_2 _for 4 h at 4°C before the measurements were made. The pH of the solution was maintained throughout the studies.

### Thermal denaturation

Thermal denaturation of MtαIPMS (0.75 μM) was monitored by change in molar ellipticity at 222 nm as function of temperature on a Jasco J810 spectropolarimeter equipped with peltier temperature controller system. The measurements were carried out in 5 mM HEPES buffer pH 7.4. Samples were heated at constant rate of 1°C/min in 1 mm cell.

### Cross linking using glutaraldehyde

The TIM barrel domain (0.2 mg/ml) was incubated with 1% glutaraldehyde (final concentration) for 20 min at 4°C followed by quenching of crosslinking reaction by addition of 2 μl of β-mercaptoethanol. The cross-linked products were concentrated by centricon (50 kDa, Millipore) and run on 8% SDS-PAGE for analysis.

## Abbreviations

α-IPMS, α-isopropylmalate synthase, α-KIV, α-ketoisovalerate, SEC, Size exclusion chromatography, ESI-MS, Electrospray ionization mass spectrometry, AcCoA, Acetyl-Co A.

## Authors' contributions

KS carried out all the experiments and the data analysis. VB conceived the study, participated in the design of the experiments and drafted the manuscript. Both the authors approved the final manuscript.

## Supplementary Material

Additional File 1 **Effect of ZnCl_2 _and CdCl_2 _on secondary structure of MtαIPMS and TIM barrel domain**. Far-UV CD spectra of MtαIPMS (Panel A and B) and TIM barrel domain (Panel C and D) in presence of increasing concentration of ZnCl_2 _(Panel A and C) and CdCl_2 _(Panel B and D). The various curves represent ZnCl_2 _and CdCl_2 _concentration of 0 mM (solid line), 0.5 mM (dashed line), 1 mM (dotted line), 2 mM (dashed dotted line), and 3 mM (small dashed line). Click here for file
