# Supplementary figures and images for: Cation induced differential effect on structural and functional properties of Mycobacterium tuberculosis α-Isopropylmalate synthase
Source: BMC Struct Biol. 2007 Jun 19;7:39. doi: 10.1186/1472-6807-7-39 (PMC1919377; doi:10.1186/1472-6807-7-39)

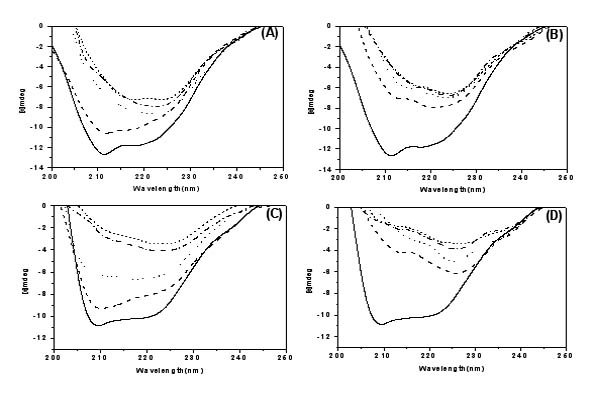

Supplement: Additional File 1 — Effect of ZnCl2 and CdCl2 on secondary structure of MtαIPMS and TIM barrel domain. Far-UV CD spectra of MtαIPMS (Panel A and B) and TIM barrel domain (Panel C and D) in presence of increasing concentration of ZnCl2 (Panel A and C) and CdCl2 (Panel B and D). The various curves represent ZnCl2 and CdCl2 concentration of 0 mM (solid line), 0.5 mM (dashed line), 1 mM (dotted line), 2 mM (dashed dotted line), and 3 mM (small dashed line). [file 1472-6807-7-39-S1.jpeg]
